# Is the obesity paradox in outpatients with heart failure reduced ejection fraction real?

**DOI:** 10.3389/fcvm.2023.1239722

**Published:** 2023-12-11

**Authors:** Nathália Felix Araujo Salvino, Lyz Tavares de Sousa, Fabio Maia Abrahao, Pedro Pimenta de Mello Spineti, Ana Luiza Ferreira Sales, Felipe Neves de Albuquerque, Marcelo Imbroinise Bittencourt, Pedro Castello Branco de Moraes, Roberto Esporcatte, Ricardo Mourilhe-Rocha

**Affiliations:** ^1^ Serviço e Disciplina de Cardiologia, Universidade do Estado do Rio de Janeiro, Rio de Janeiro, Brazil; ^2^ Complexo Hospital Americas - Vitória e Samaritano - Barra da Tijuca, Rio de Janeiro, Brazil; ^3^ Universidade Estácio de Sá, Departamento de medicina, Rio de Janeiro, Brazil

**Keywords:** obesity paradox, heart failure, obesity, MAGGIC score, heart failure reduced ejection fraction

## Abstract

**Background:**

The obesity occurrence has achieved epidemic levels worldwide and several studies indicate a paradoxical similarity among obesity and the prognosis in heart failure (HF). The primary objective was to understand the association between body mass index (BMI) and heart failure with reduced ejection fraction (HFREF) of ischemic etiology in outpatients, using mortality as a parameter. The secondary objectives were to determine the differences in HF functional class, pharmacological therapy and evaluate the prognostic value of MAGGIC Score in this population.

**Methods:**

We analyzed 1,556 medical records from the HF outpatient clinic of a quaternary hospital and 242 were selected according to the criteria. Most were male, average age 62.6 (56–70), BMI 18.5–24.9 = 35.1%, 25–29.9 = 37.2%, 30–34.9 = 17.8%, 35–39.9 = 7%; BMI <18.5 and >40 groups were eliminated from the central analyzes because of scarce testing.

**Results:**

BMI 30–34.9 and BMI 18.5–24.9 had the best prognosis, BMI 25–29.9 had an average performance, and BMI –39.9 group provided the worst outcome (*p* = 0.123). In the subcategory analysis, BMI 30–34.9 group had a better prognosis compared to the BMI 35–39.9 group (*p* = 0.033). In the multivariate analysis The MAGGIC score was not able to foretell mortality in this population according to BMI.

**Conclusion:**

In not hospitalized patients with HFREF of ischemic etiology, obesity was not a protective factor.

## 1. Introduction

The obesity occurrence has achieved epidemic percentages worldwide and is linked to variations in cardiovascular composition and function. A high BMI is a separate risk factor for HF (1, 2). An important study indicated that for every 1 kg/m^2^ up in the body mass index (BMI), the risk of HF enhances up by 7.0% in women and by 5.0% in men (3).

It is also recognized that in obese people the risk of anticipated death is doubled in comparison to non-obese people and the chance of death from cardiovascular disease is increased by five times (4) and is still associated to a bigger incidence of atrial and ventricular arrhythmias and sudden death (4, 5). In addition, obesity brings consequences, such as hypertension, diabetes, dyslipidemia, and obstructive sleep apnea, raising even more the probability of cardiac involvement.

More recently, chronic inflammation has been shown to have the potential to exacerbate HF. The inflammasome may play a central role in this case and in turn affecting HF progression (6). Thus, the inflammatory state caused directly by obesity, as well as indirectly through hyperlipidemia and hyperglycemia, can activate a highly inflamed NLRP3 with the potential to cause organic dysfunction, myocardial injury (7).

In contrast, other studies have described a paradoxical relation between a high BMI and the prognosis of patients with coronary artery disease and HF, called the obesity paradox (3, 5, 8–13). According to this theory, contrary to expectations, overweight or patients with obesity have a better prognosis in heart diseases when related to low or normal weight patients (3, 8, 9, 12, 14).

That way, although the obesity paradox may result from statistical biases, the possibility of a beneficial effect related to obesity must be taken in consideration (10).

## 2. Methods

This work consists of an observational, retrospective study, unicentric, conducted through the analysis of medical records of patients followed at the HF outpatient clinic of a quaternary hospital. Medical records with initial consultation from November 1997 up to August 2019 were evaluated, with a maximum follow-up period of 10 years and a minimum of 6 months being determined.

This study complied with the Declaration of Helsinki. The need for written informed consent was waived. The study was approved by the Research Ethics Committee of Hospital Universitario Pedro Ernesto through the Brazil platform. Approval Number: CAAE: 00406818.6.0000.5259.

### 2.1. Inclusion criteria

Age over or equal to 18 years old and under or equal to 90 years old; and presence of HFREF of ischemic etiology; and presence of anthropometric data (weight and height) described in the first consultation in the HF Clinic; and at least one consultation between 2010 and August 2019.

The Universal Definition of HF (15), as well as the Update of Emerging Topics of the Brazilian Guidelines on HF (16) classify this condition according to the ejection fraction: heart failure preserved ejection fraction (HFPEF) ≥50%; HFREF <40%; HF with slightly reduced ejection fraction 40–49%.

In this study, we considered HFREF of ischemic etiology, those with an ejection fraction <50% and a previous history of acute myocardial infarction or with coronary angiography with obstructions greater than 50% in the left main coronary artery or obstructions greater than 70% in the other arterial, proximal or middle segments; or functional tests with criteria for ischemia (myocardial scintigraphy, myocardial resonance image, stress echocardiogram or exercise test); or patients who present with segmental changes in echocardiography that respect the coronary territory associated with high clinical probability of coronary artery disease.

### 2.2. Exclusion criteria

Patients with liver cirrhosis, decompensated or not; with chronic kidney disease in renal replacement therapy; with active cancer, characterized by a diagnosis of cancer for less than 5 years or for more than 5 years without evidence of the cure; with an earlier history of heart or kidney transplantation.

### 2.3. Variables analyzed

Demographic, anthropometric, clinical, laboratory, echocardiographic, therapeutic, and prognostic variables were entered into the database.

To define and classify the nutritional status, the European Guideline for the Treatment of Obesity in Adults was chosen (17). The grade I obesity group was the reference group for comparing demographic and clinical characteristics between groups, considering the hypothesis that obesity is related to more meaningful mortality in HF.

### 2.4. Statistical analysis

In the descriptive analysis, the categorical variables were affected through their frequency. Continuous variables were perforated by their median, and 25th and 75th percentiles, as they presented a non-normal distribution. The normality of the continuous variables was assessed through their distribution pattern on the histogram and the Kolmogorov–Smirnov test.

To compare the different extracts of nutritional classification, the categorical variables were compared using the chi-square test and Fisher's exact test. The continuous variables were compared using the Mann–Whitney *U*-test. Kaplan–Meyer curves, stratified by nutritional status, were constructed to assess survival. The curves were compared using the log-rank test.

Cox analysis was performed to assess the prognostic value of each strata of nutritional status in relation to survival.

The IBM SPSS 27.0 program for Windows was used for statistical analysis. The significance level adopted was 5%.

## 3. Results

### 3.1. Study population and baseline characteristics

**Figure 1:**
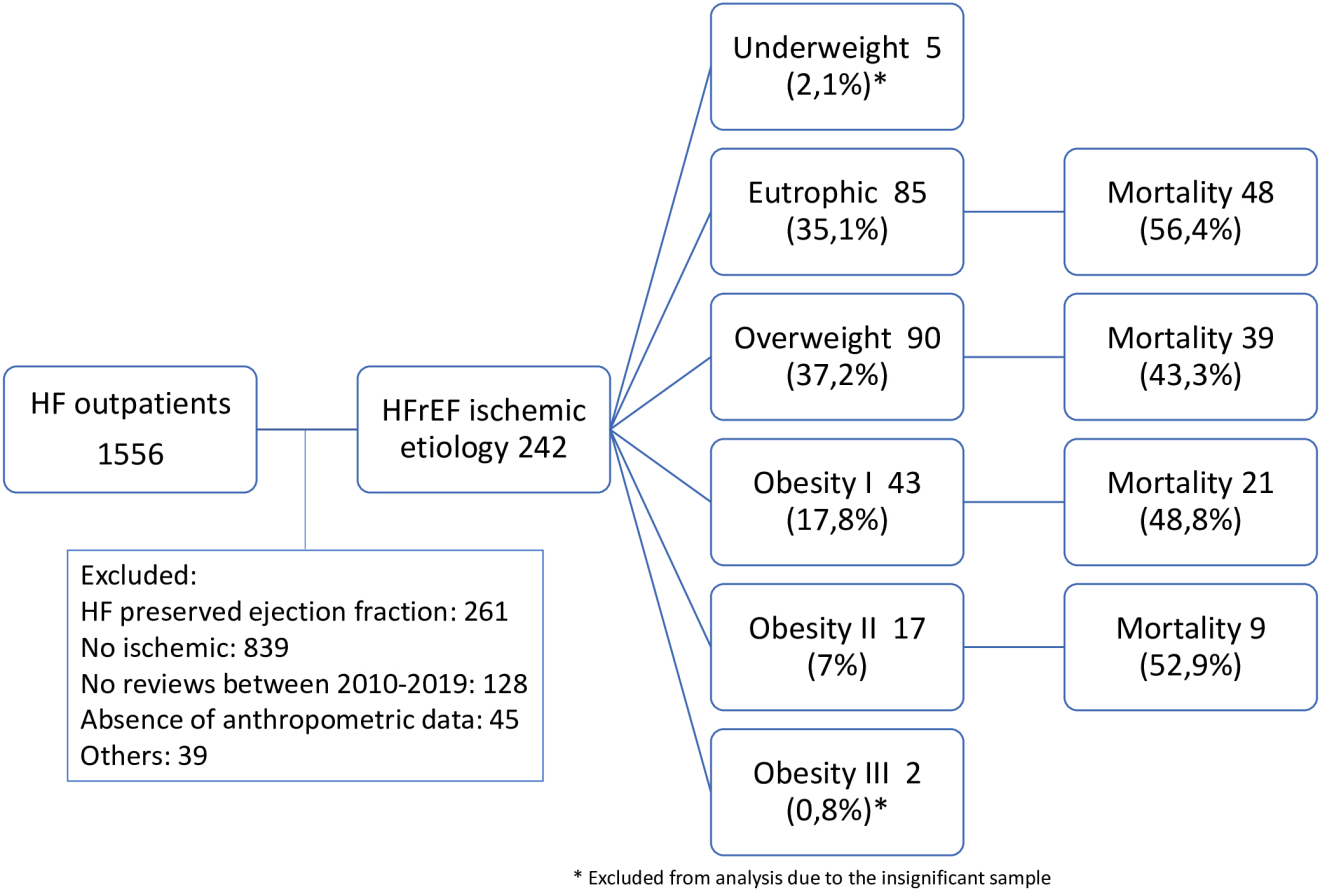
Patient selection/exclusion flow according to nutritional class and mortality.

In this study, 1,556 records from the HF outpatient clinic were analyzed, of which 242 patients were included (Figure 1). Characteristics of the population: 71% were male, the median time of HF was 12.1 (2.9–40.9) months. The study subgroups were constituted of 5% underweight, 35.1% eutrophic (64.9% male gender), 37.2% overweight (75.6% male gender), 17.8% obesity grade I (69.8% male gender), 7% obesity grade II (64.7% male gender) and 0.8% obesity grade III. The underweight and obesity grade III groups were eliminated from the central analyzes because of scarce testing. The sample clearly showed a marked male predominance at all BMI levels. The standard information of the research population, also divided by subgroups, are summarized in Table 1.

**Table 1:** General characteristics of the whole population.

	General population	Eutrophic	Overweight	Grade I obesity	Grade II obesity
Number (*n*, %)	242	85 (35.1%)	90 (37.2%)	43 (17.8%)	17 (7%)
Age (years)	62.6 (64–88)	66.2 (58.7–74)	61.5 (55.9–69.8)	61.4 (53.1–69.9)	61.1 (56.9–66.5)
Male sex (*n*, %)	172 (71.1)	59 (64.9)	68 (75.6)	30 (69.8)	11 (64.7)
Hypertension (*n*, %)	230 (95)	78 (91.8)	87 (96.7)	42 (97.7)	16 (94.4)
Diabetes mellitus (*n*, %)	117 (48)	29 (34.1)*	46 (51.1)	26 (60.5)	13 (76.5)
Smoker (*n*, %)	33 (13.6)	14 (16.5)	12 (13.3)	6 (14)	1 (5.9)
Alcoholism (*n*, %)	44 (18.2)	15 (17.6)	18 (20)	6 (14)	5 (29.4)
COPD (*n*, %)	11 (4.5)	4 (4.7)	3 (3.3)	3 (7)	1 (5.9)
Atrial fibrillation (*n*, %)	54 (22.3)	20 (23.5)	18 (20)	7 (16.3)	6 (35.3)
CKD (*n*, %)	66.5 (51.7–84.6)	63.9 (44.7–82.8)	70.4 (50–84)	62.5 (39.4–75.5)	62.9 (11.4–88.9)
Ejection fraction (%)	35 (26–41)	34 (25–41.7)	35 (26–40)	36 (29–42.5)	37.5 (128–44.7)
NYHA I (*n*, %)	26 (10.7)	7 (8.2)	12 (13.3)	6 (14)	1 (5.9)
NYHA II (*n*, %)	115 (47.5)	40 (47.1)	42 (46.7)	22 (51.2)	6 (35.3)
NYHA III (*n*, %)	77 (31.8)	30 (35.3)	29 (32.2)	9 (20.9)	7 (41.2)
NYHA IV (*n*, %)	24 (9.9)	8 (9.4)	7 (7.8)	6 (14)	3 (17.6)
ARB/ACE-I/ARNI (*n*, %)	207 (85.5)	70 (82.3)	82 (91.1)	35 (81.4)	14 (82.4)
MRA (*n*, %)	118 (48.8)	39 (45.9)	46 (51.1)	20 (46.5)	10 (58.8)
Beta blockers (*n*, %)	202 (83.5)	70 (82.4)	74 (82.2)	36 (83.7)	15 (82.2)
Mortality (*n*, %)	121 (50)	48 (56.4)	39 (43.3)	21 (48.8)	9 (52.9)
MAGGIC score	21 (16–27)	22 (17–27)	21 (15–28)	21 (17–25)	19 (14–28)

Continuous variables are expressed as medians. Categorical variables are expressed as total number (%). CKD, chronic kidney disease; COPD, chronic obstructive pulmonary disease; ARB, angiotensin receptor blockers; ACE-I, angiotensin-converting enzyme inhibitors; ARNI, angiotensin receptor-neprilysin inhibitor; MRA, mineralocorticoid receptor antagonist; NYHA, New York Heart Association.

* *p* < 0.05.

NYHA (New York Heart Association) functional class is known to be a strong prognostic marker (18), most of the patients were in NYHA II, except for the obesity grade II group, which showed a predominance of patients in functional class III. This characteristic can represent a confounding factor on the outcome evaluation. Once the obesity group grade I was chosen as the reference for analysis in relation to the other groups, the Fischer test was performed, and did not establish a statistically notable difference in comparison to functional class.

In the obesity group grade II, we observed a more elevated incidence of alcoholism, atrial fibrillation, and diabetes. All these variables can adversely influence the prognosis. Hence, a comparison was performed using the chi-square test to assess the statistical importance of these data. However, except for the difference in the number of diabetics between eutrophic and obesity grade I groups, the other factors were not statistically relevant, taking the obesity group grade I as a reference. Regarding the extent of LV systolic dysfunction, specific four main groups under analysis had similar profiles, with a predominance of severe systolic dysfunction. Regarding laboratory variables: sodium, potassium, hemoglobin and creatinine clearance, there was no striking variation between the groups analyzed. Regarding the established therapy, the four main groups of analysis used proportionally the main prognostic modifying drugs.

### 3.2. Primary endpoint: for all causes mortality

**Figure 2:**
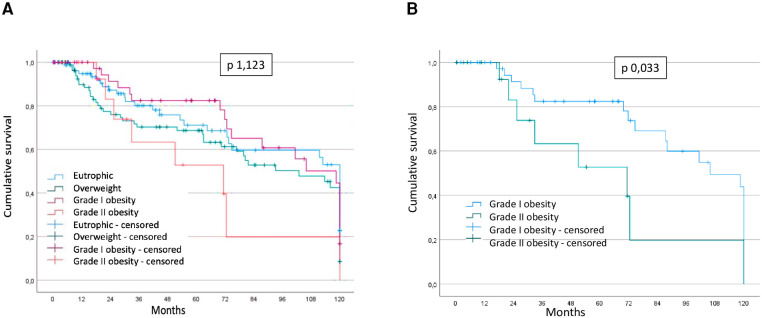
(**A**) Survival according to nutritional classification and (**B**) survival grade I obesity versus grade II obesity.

Among the analyzed groups, the worst survival was in the obesity group grade II (*p* = 0.123) (Figure 2A). In the subgroup evaluation, using the obesity group grade I as a reference, this showed better survival than the obesity group grade II (*p* = 0.033) (Figure 2B) and, there was no statistically significant difference between obesity grade I and overweight group, and obesity grade I and eutrophic group.

Survival analysis using COX univariate analysis comparing the study groups with the grade I obesity group based on nutritional classification show: eutrophic (OR 0.12; CI 0.02–0.08; *p* = 0.037), overweight (OR 0.15; CI 0.02–1.09; *p* = 0.06) and obesity II (OR 0.22; CI 0.03–1.76; *p* = 0.15) (Supplementary Material).

### 3.3. Secondary endpoints

Specific therapy and HF severity did not impact the prognosis according to nutritional status in HF.

**Figure 3:**
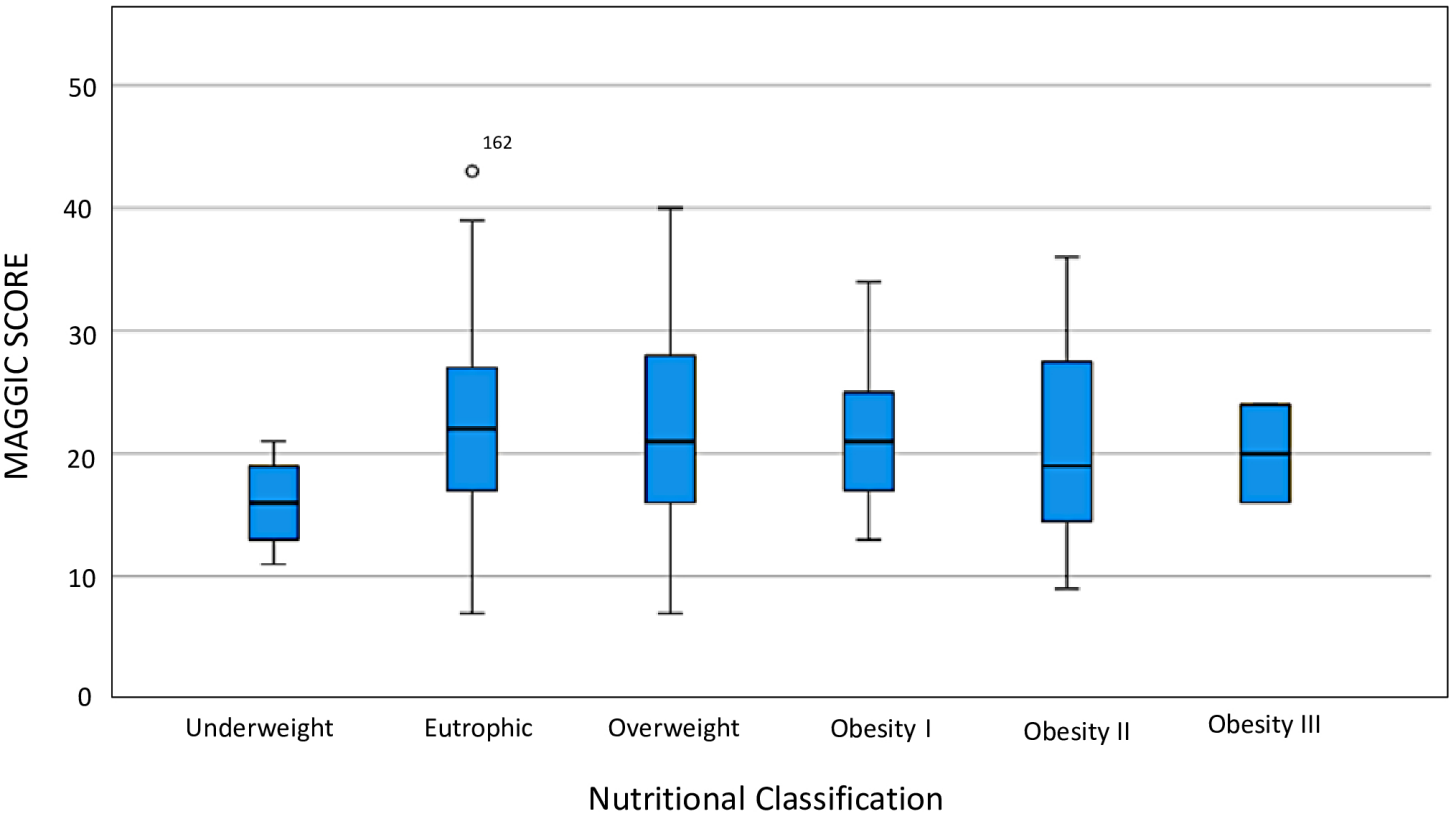
MAGGIC score according to nutritional classification.

There was no difference in the MAGGIC score means stratified according to nutritional status (Figure 3). MAGGIC score did not predict mortality in our sample in the univariate Cox analysis (p0,995). Therefore, we did not perform a multivariate Cox analysis.

## 4. Discussion

The result of this study is against of the theory of the paradox of obesity in patients with HFREF of ischemic etiology since obese patients did not experience a better outcome than eutrophic patients. Furthermore, in the subgroup analysis, using obesity I group as reference there was no statistically striking variation when compared to overweight and eutrophic groups. However, having grade II obesity was worse than having grade I obesity, reinforcing the lack of benefit, in terms of survival, related to a higher BMI.

On the other hand, a study that pursue to determine if BMI delivered a differential impact on the survival of women compared to men with advanced systolic HF reviewed 3,811 patients with HFREF. The outcome was mortality all-cause. Unadjusted data demonstrated the obesity paradox throughout overall survival in HF; however, this phenomenon disappeared after adjustment for confounding factors (age, race, ischemic etiology, NYHA functional class, pharmacotherapy, diabetes, smoking, hypertension, hypercholesterolemia, peak VO_2_, subsequent transplantation or left ventricular assist device and others). Thus, overweight, and obese men had more elevated adjusted mortality compared to normal-weight men, while a BMI in the overweight selection was connected to a significant survival benefit in women, also weakening the paradox theory as well as our study (19).

From another perspective, a recent study evaluates the influence of sex, BMI and age or left ventricular global longitudinal strain in the association with mortality and the obesity paradox, and indicates that sex, aging and myocardial dysfunction can affect its magnitude, however it is important to say that the patients analyzed are from East Asia, so it is uncertain whether the results can be generalized to other ethnicities (20).

Similarly, another study that evaluated the obesity paradox in stable chronic HF patients in a cohort of 1,790 patients reported that this phenomenon does not seem to exist after adjusting for confounding factors such as age, gender, and functional class. Furthermore, the same study emphasizes that the laboratory marker NT pro BNP remains a prognostic marker independently of the presence of obesity (21). From our sample, in which 71% of patients were male, there was no statistical significance among the possible confounding factors, and the result was also unfavorable to the obesity paradox. This reinforces the possibility that this theory is the result of statistical bias.

Besides, a cohort with a total of 5,819 patients with chronic HF were separated into four groups based on BMI. Mean age was 65 ± 12 years, with most males, ischemic HF and HFREF, similar as our population. The frequency of all-cause mortality or HF hospitalization was worse in the lowest two BMI groups. Nevertheless, this impact was seen in patients older than 75 years or patients who have no less than one relevant co-morbidity. When other points were evaluated, like, medications and laboratorial findings, the prognostic impact of nutritional classification was absent even in the elderly group with co-morbidity. Therefore, this study suggests that prominent levels of BMI do not represent a protective effect in patients with chronic HF (22).

In another perspective, several studies in favor of the obesity paradox in HF have been conducted in hospitalized patients (23, 24). A pooled study of two monitoring studies of multicenter HF investigated the impact of obesity in patients hospitalized for HFREF (ejection fraction ≤45%). The selected patients, 3,145 (1,824 men), were divided into two groups, a high BMI group and a normal BMI group. In the high BMI group, the 1-year mortality rate was lower among men even after adjustment for clinical characteristics (25). However, it is worth remembering that most patients hospitalized for HF present congestion, which can lead to an overestimation of body weight due to fluid overload. In our study, which was conducted exclusively in outpatients, and therefore more compensated patients, this was not confirmed. Furthermore, a recent review article on this subject, which included 79 publications, suggests that the lack of data on body composition, visceral fat, sarcopenic obesity, muscle and cardiorespiratory fitness can significantly influence the results, leading to incorrect conclusions (26).

In the other hand, a recent meta-analysis evaluating inpatients with acute coronary syndrome and noted that obesity is moderately associated with traditional cardiovascular risk factors and fewer negative outcomes, however, a minority of the patients studied had previous HF, which prevents a comparative analysis with our study, which was carried out exclusively in outpatients with HFREF of ischemic etiology (27).

It is important to mention that a recent study included more than 47,000 patients with HF and found that there is a *U*-shaped relationship between BMI and long-term all-cause mortality, since when comparing people with normal weight with people overweight, grade I obesity and grade II obesity, these showed a lower risk of death. On the other hand, people who are underweight have an increased risk of death. Furthermore, grade III obesity was associated with increased risk of all-cause mortality compared to being overweight. However, the population of this analysis was more heterogeneous than ours, as the inclusion criteria do not specify whether it is HFREF or HFPEF and as well as cause of HF (28).

In contrast, many studies have suggested that the presence of the obesity paradox is influenced by the etiology of HF. In a recent analysis, a retrospective multicenter study compared the presence of this phenomenon in patients with acute HF with and without a previous history of coronary artery disease and this phenomenon was not confirmed in the presence of coronary artery disease associated with HF (29), a result like in this cohort, which is also composed of patients with HF of ischemic etiology, but on an outpatient basis.

Another study similarly assessed whether there is an influence of the etiology of HF (ischemic vs. non-ischemic) in relation to the effect of BMI on the prognosis of outpatients. Among 504 patients, 59% had HF of ischemic etiology. The median left ventricular ejection fraction was 30% (23%–39.7%). NYHA functional class II (51%) and III (42%). Patients were segmented according to BMI. In this study, mortality differed significantly between BMI strata in non-ischemic patients, but not in ischemic patients. So, the obesity paradox was not showed in HF of ischemic etiology, like in our sample (30).

Likewise, a recent study conducted with 5.155 outpatients with HF with varying degrees of ejection fraction evaluated the impact of obesity on HF. As a result, both overweight and mild-to-moderate obesity were linked with a better outcome in non-ischemic, but not in ischemic HF (31). In our sample, composed of outpatients with a decreased ejection fraction of ischemic etiology, similarly, having obesity was not a protective factor, confirming the findings of this large study.

Besides that, several topics in the analysis of the obesity paradox must be evaluated. In numerous studies, high BMI individuals were characterized by younger age, fewer arrhythmias, less anemia, better left ventricular systolic function, and better renal function (4). In our population, the age and renal function were comparable and, using grade I obesity group as reference, the functional class difference and atrial fibrillation difference were not statistically relevant.

From another perspective, diabetes mellitus (DM) is highly prevalent among obese patients with HF and is related with unfavorable prognosis. A High BMI increases the risk of cardiovascular complications and mortality in DM, and weight loss is indicated to improve glycemic control and other cardiovascular risk factors. Thus, a study that analyzed patients with mild-to-moderate chronic HF showed that in the presence of DM, obesity does not confer any paradoxical benefit on survival. However, whether intentional weight reduction can promote benefits in these patients needs more investigation in future studies (32). Furthermore, a study that evaluated 2,527 outpatients with HF (1,102 with type 2 diabetes) demonstrated the paradox of obesity in patients with HF in the absence of type 2 diabetes; however, type 2 diabetes removed this phenomenon (33). In our sample, comprising 48% of patients with diabetes, there was also no evidence of benefits in relation to high BMI.

Nevertheless, a recent study assessed whether the percentage of body fat estimated using equations reveals associations with a clinical outcome and biomarkers of HF. The median body fat percentage was 26.9% (Jackson–Pollock equation) and 28.0% (Gallagher equation). Patients in the first tertile of body fat percentage had the least favorable outcomes, and patients in the second and third tertile had comparable survival. Both BMI and body fat percentage were opposite predictors of NT-proBNP, but not high-sensitivity troponin T. Therefore, in obese patients (BMI >30 kg/m^2^, third tertile of percent body fat), high-sensitivity troponin T and soluble suppression of tumorigenesis-2 independently predicted the clinical outcome, which was not the case for NT-proBNP. However, although these results provide a very convincing relationship between body fat percentage and survival in HF, it is relevant to note that these data were estimated using equations validated in healthy individuals. Additionally, the first tertile included patients with low weight, a condition known to be connected with a worse prognosis in HF (34).

In the multivariate analysis, the MAGGIC score was not able to predict mortality in this population, which may represent an effect of longer follow-up time or small N. From another perspective, a sub analysis of the MAGGIC study published in 2014 assessed the obesity paradox by separating patients with HF into HFREF and HFPEF, analyzing the BMI of 23,967 patients and, in both groups, mortality was lower among patients with BMI between 30 and 34.9 kg/m^2^ (35). Nonetheless, this analysis did not subdivide the HFREF group into patients with ischemic vs. non-ischemic heart disease, which would favor a more precise understanding of the impact of etiology in this context.

In this manner, although the paradox of obesity in heart disease is an extensively studied concept, it is still subject to questioning. In this analysis, consisting of outpatients and, therefore, more compensated, this theory was not confirmed. Hence, this analysis contributes to demystify this theory, which biologically does not make sense to patients with HFREF of ischemic etiology. As a conclusion, BMI does not represent a reliable prognostic marker in patients with HFREF, given the variation in the volume status of this patient profile. Future research should include body composition measurements, such as waist circumference and DEXA or bioimpedance analysis, for better analysis.

This study contains many limitations, including being a retrospective, observational, and a single-center study, conducted in a public hospital; thus, the characteristics and data obtained may not reflect the profile of the disease in other countries. Furthermore, the majority of members are male, which may limit our conclusions regarding women. On the other hand, the small sample size is a major limitation, particularly the exceedingly small number of patients with grade II obesity.

Additionally, the lack of knowledge of the volume status when measuring weight remains a limiting factor in the obesity paradox, as the presence of edema in this profile of the patient is frequent, a factor with a significant influence on weight, and thus with an impact on BMI.

In conclusion, in outpatients with HFREF of ischemic etiology, having obesity was not a protective factor, not confirming the theory of the “obesity paradox”. Furthermore, no nutritional classification range provided a more beneficial prognosis in the study population focused on survival. The presence of comorbidities, severity of HF and specific therapy did not impact the prognosis according to nutritional classification. The MAGGIC score failed to foresee death in this sample, most likely due to the effect of small number of patients or longer follow-up time.

## Data Availability

The datasets presented in this study can be found in online repositories. The names of the repository/repositories and accession number(s) can be found below: http://www.bdtd.uerj.br/handle/1/18538.

## Supplementary material

Supplementary Material Survival analysis using COX univariate analysis comparing the study groups with the grade I obesity group based on nutritional classification.
