# Supplementary figures and images for: Is the obesity paradox in outpatients with heart failure reduced ejection fraction real?
Source: Front Cardiovasc Med. 2023 Dec 11;10:1239722. doi: 10.3389/fcvm.2023.1239722 (PMC10750383; doi:10.3389/fcvm.2023.1239722)

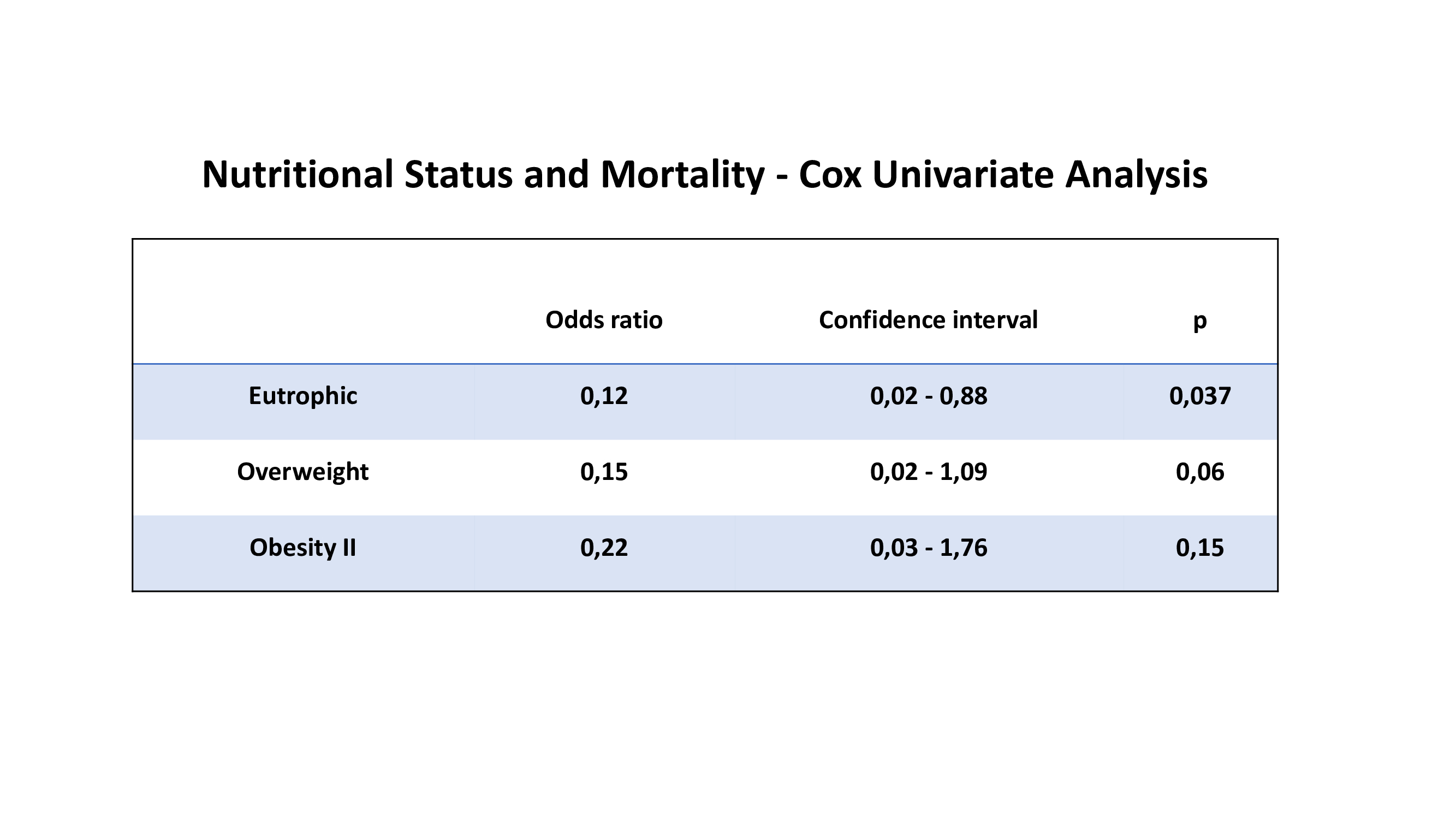

Supplement: Supplementary Material — Survival analysis using COX univariate analysis comparing the study groups with the grade I obesity group based on nutritional classification. [file Image1.jpeg]
